# Supplementary material for: Microscopic insight into non-radiative decay in perovskite semiconductors from temperature-dependent luminescence blinking
Source: Nat Commun. 2019 Apr 12;10:1698. doi: 10.1038/s41467-019-09640-w (PMC6461618; doi:10.1038/s41467-019-09640-w)
Supplement: Supplementary file 1 — Supplementary Information [file 41467_2019_9640_MOESM1_ESM.pdf]

Supplementary Information:

Microscopic insight into non-radiative decay in perovskite semiconductors from temperature-dependent luminescence blinking

Marina Gerhard, Boris Louis, Rafael Camacho, Aboma Merdasa, Jun Li, Alexander Kiligaridis, Alexander Dobrovolsky, Johan Hofkens and Ivan G. Scheblykin

Table of Figures

Supplementary Figure 1: Overview PL image 3
Supplementary Figure 2: Temperature-dependent PL intensity in individual crystals 4
Supplementary Figure 3: Measurements of absolute blinking amplitudes. 5
Supplementary Figure 4: Residence time analysis. 6
Supplementary Figure 5: Switching rates of different crystals 7
Supplementary Figure 6: Comparison between experiment and simulation for some crystals 8
Supplementary Figure 7: Measured and simulated features extracted from the blinking transients ... 9
Supplementary Figure 8: Histogram-type illustration of the simulation parameters. 9
Supplementary Figure 9: Average number of active quenchers 10
Supplementary Figure 10: Absorption and emission spectrum at room temperature 11
Supplementary Figure 11: Temperature dependent PL spectra 12
Supplementary Figure 12: Exemplary SEM data 13
Supplementary Figure 13: SEM images (left) and blinking traces (right) recorded for two crystals 13
Supplementary Figure 14: Estimation of the crystal size 14
Supplementary Figure 15: Estimation of the excitation fluence 15
Supplementary Figure 16: Estimated PLQY at different temperatures 17
Supplementary Figure 17: PL intensity as function of ensemble-averaged excitation fluence 18
Supplementary Figure 18: Power- and temperature dependent CDF plots of relative blinking amplitudes 19
Supplementary Figure 19: Switching behavior as function of excitation density recorded at 260 K ... 19
Supplementary Figure 20: Room temperature PL dynamics. 20
Supplementary Figure 21: PL decay transients for different temperatures 21
Supplementary Figure 22: Estimation of the fraction of free charges 22
Supplementary Figure 23: Connection between PL dynamics and PL intensity in the blinking transients 24
Supplementary Figure 24: Illustration of different quenching mechanisms 25
Supplementary Figure 25: Lifetime intensity correlation plots 26
Supplementary Figure 26: Number of crystals above a pre-defined SNR of 2 27
Supplementary Figure 27: Estimation of $\kappa(T)$ 29
Supplementary Figure 28: Time-averaging effect for different switching times 30

Table of Contents

Supplementary Note 1: Scanning electron microscopy and correlation with luminescence 13
Supplementary Note 2: Estimation of the excitation density 15
Supplementary Note 3: Estimation of the photoluminescence quantum yield (PLQY) 17
Supplementary Note 4: Excitation power dependent measurements 18
Supplementary Note 5: Time-resolved photoluminescence (TRPL) 20
Supplementary Note 6: Estimation of the fraction of free charges 22
Supplementary Note 7: Correlation between PL intensities and PL lifetime 24
Supplementary Method 1: Selection of crystals for blinking analysis 27
Supplementary Method 2: Simulation details 28
Supplementary References 32

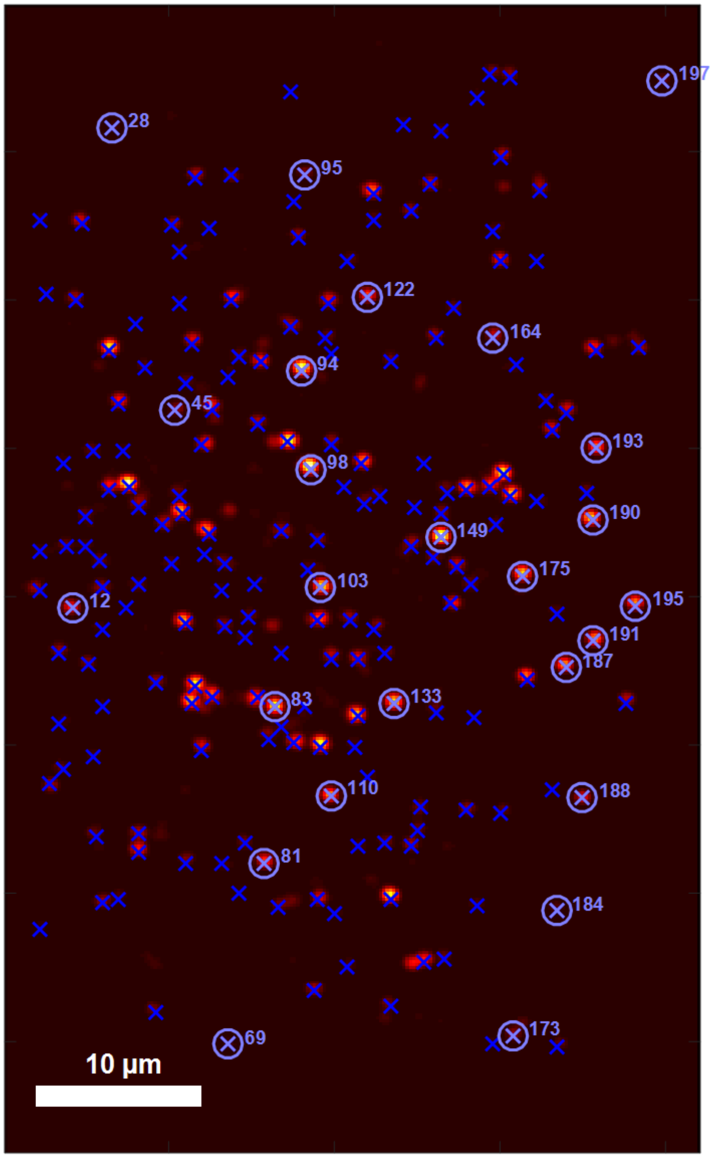

Supplementary Figure 1: Overview PL image. The image was obtained from time-averaging a PL movie recorded at 140 K. Crosses correspond to the positions of all crystals that were localized in the average image. The numbered positions highlight the crystals that fulfilled the selection criteria described in Supplementary Method 1 throughout the whole temperature range for both heating and cooling cycle. For this subset of crystals, we performed fits of the temperature dependent PL intensity, as detailed in Supplementary Figure 2.

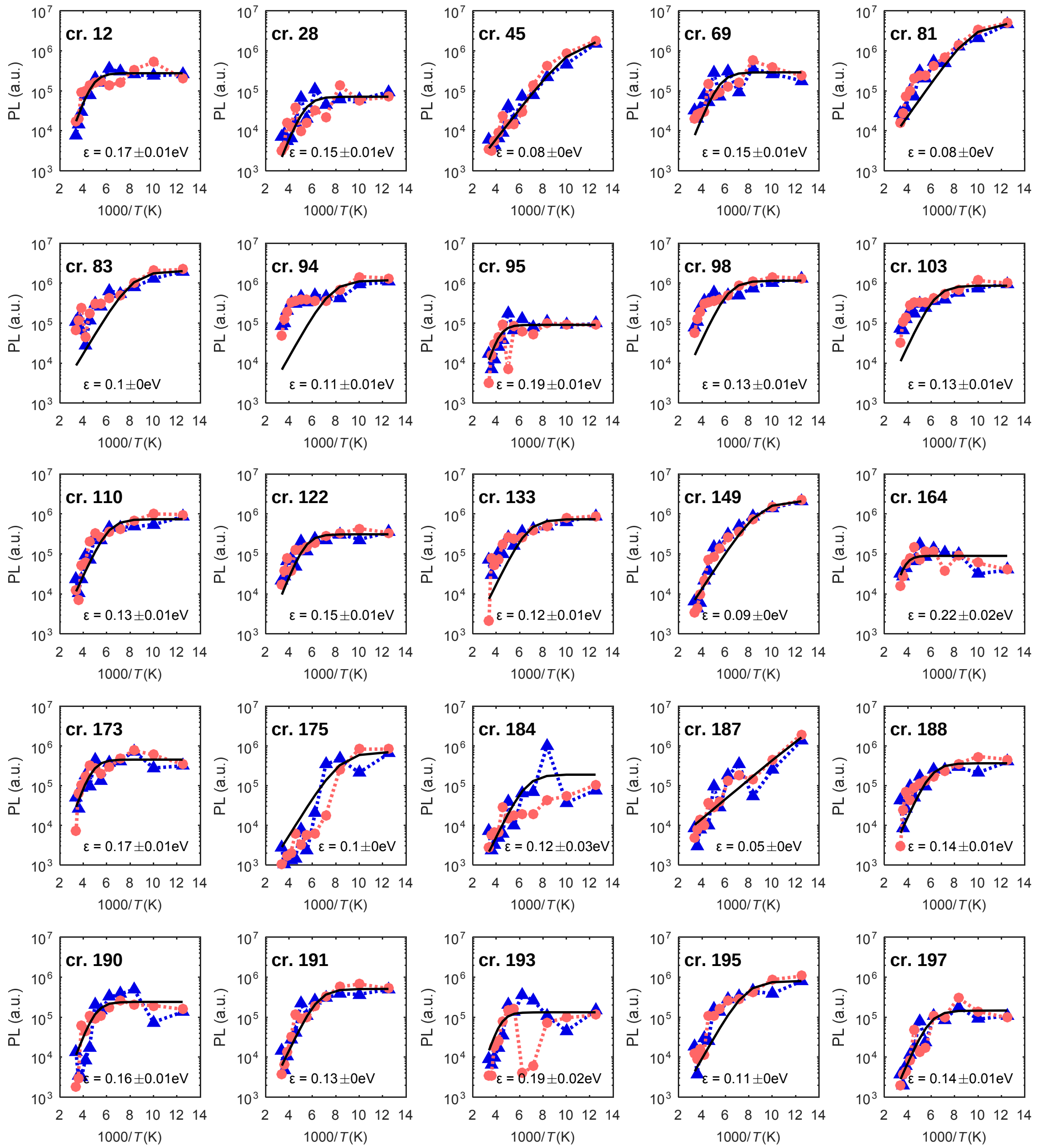

Supplementary Figure 2: Temperature-dependent PL intensity in individual crystals. We analyzed a group of 25 crystals that fulfilled our pre-defined selection criteria over the whole temperature range. Blue triangles denote the cooling and red circles the heating cycle. The data was fit by an activation law (black curves, see Equation 1 in the main text) and the activation energies ε obtained from this fitting are displayed in the respective panels. In some cases, the fit gives good agreement with the recorded intensity curves, but in many other cases, remarkable deviations from an activation model with only one energetic barrier become apparent. Moreover, the fits reveal a broad distribution of activation energies, which is presented in the histogram in Figure 1(k) in the main text. Overall, these results support the idea that the PL enhancement upon cooling depends strongly on the type and amount of defects in the material.

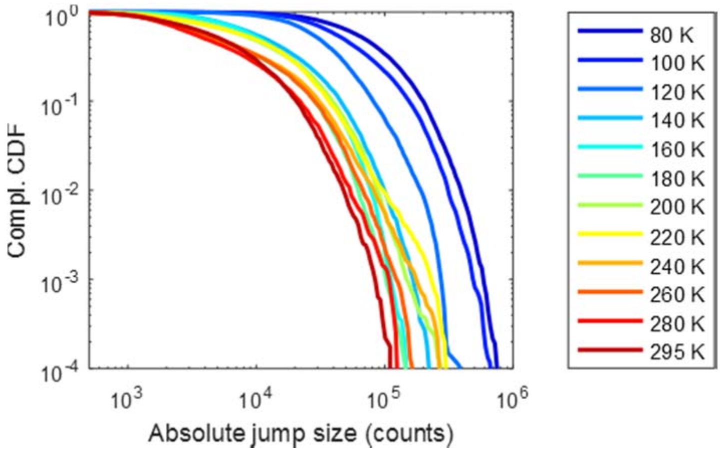

Supplementary Figure 3: Measurements of absolute blinking amplitudes. The plot shows the complementary cumulative distribution functions (CDF) of absolute intensity jumps extracted from the blinking transients at different temperatures. For meaningful analysis, the PL intensity was divided by the local excitation power for each crystal. Overall, we observe an increase of the absolute blinking amplitudes by a factor of about 10, when the sample is cooled from room temperature to 80 K. This increase is, however, smaller than the overall ensemble averaged enhancement of the PL intensity (factor 100). Thus, the fluctuating part of the PL emission, which is described by the plot of the relative blinking amplitudes in the main paper, decreases upon cooling.

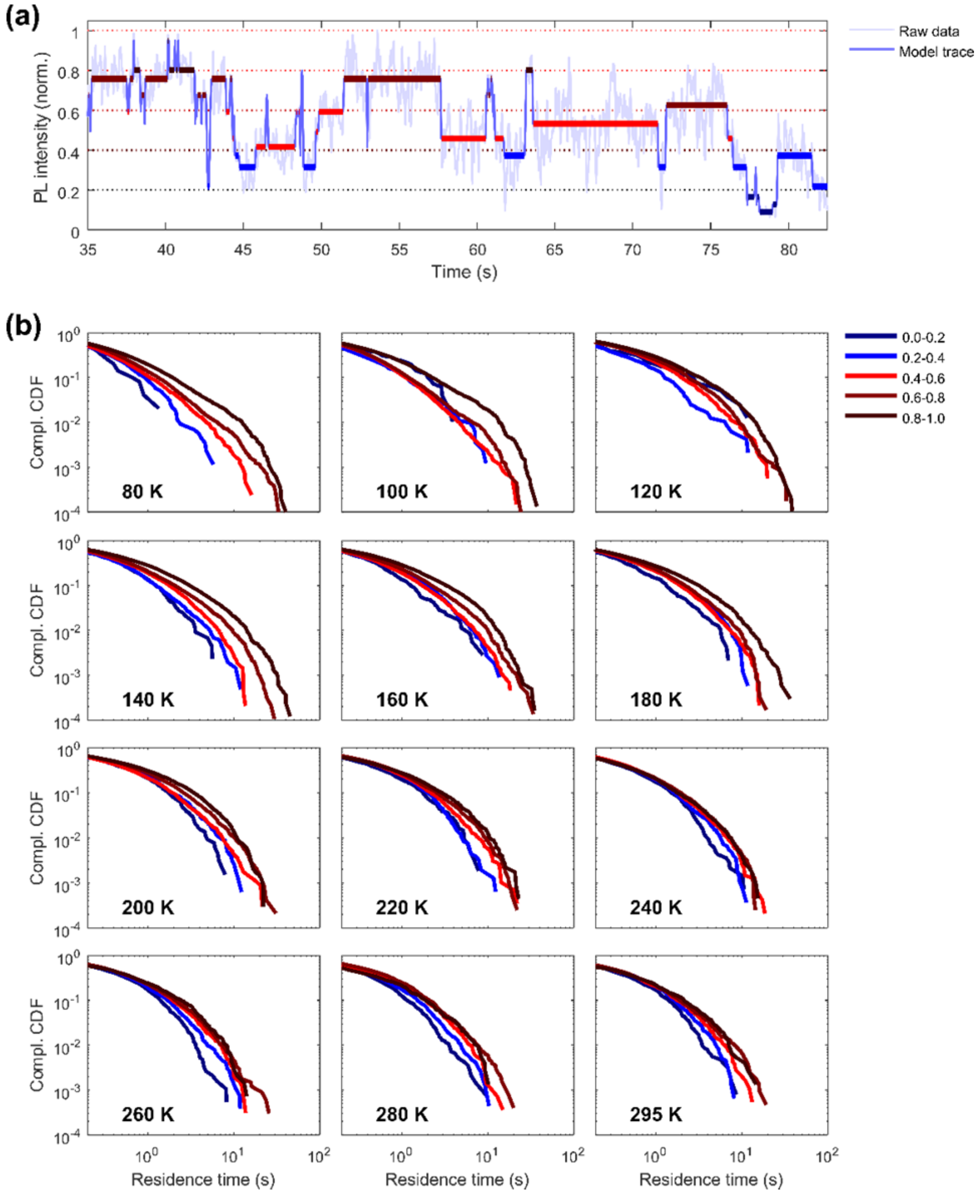

Supplementary Figure 4: Residence time analysis. A residence time denotes a time period, during which the crystal intensity stays at a particular intensity level. Due to the presence of several quenchers in one crystal, it is not possible to make an unambiguous statement about the underlying time periods, during which a quencher is active or passive. Thus, instead of performing a conventional 'ON' and 'OFF' time characterization, we analyze how these residence times are correlated with the relative brightness of the corresponding intensity levels. This analysis helps us to draw further conclusions about the underlying switching times. To exemplify the method, panel (a) shows raw data with a model trace (blue) and the extracted residence times indicated as thick lines. The raw data is normalized by the maximum intensity and residence times in different intensity ranges relative to the maximum intensity are analyzed separately, as indicated by the different colors. In (b), plots of the complementary cumulative distribution function (CDF) of residence times extracted from the whole ensemble of crystals in different intensity intervals are presented. In the low temperature data, we note that the residence times at low intensity levels tend to be shorter than the time periods spent at bright intensity levels. This discrepancy decreases with increasing temperature. The residence time data thus gives support for the hypothesis that with decreasing temperature, the time periods, during which the quenchers are passive, become longer.

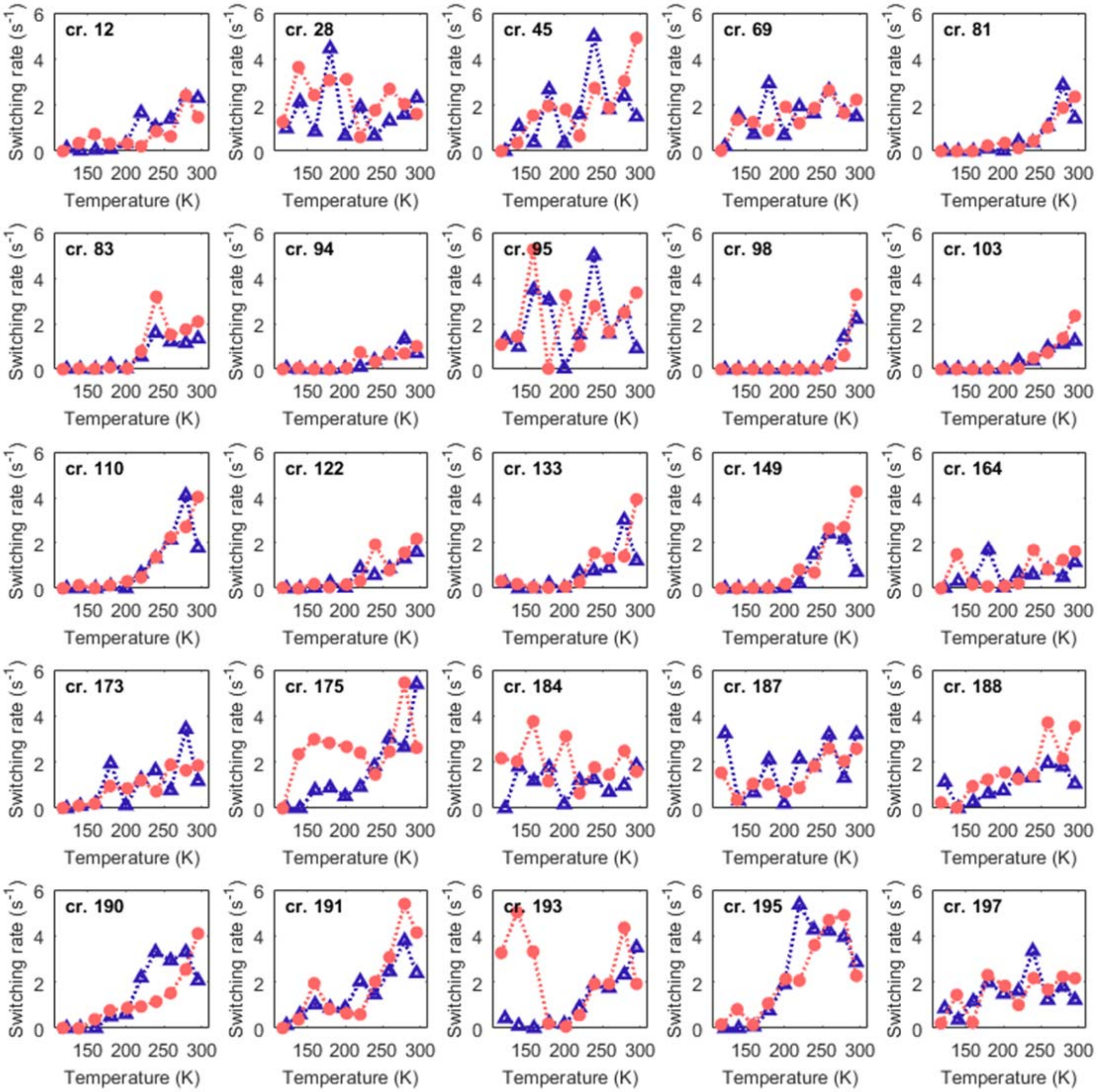

Supplementary Figure 5: Switching rates of different crystals. Blue triangles denote the switching rates extracted for cooling and red circles the rates for heating of the crystals, demonstrating reproducible behavior. For the counting of switches, we considered only blink events exceeding a change of 20 % of the maximum amplitude. To obtain a switching rate, the number of events counted in the blinking transients was divided by the measurement time of 100 s. The selected crystals correspond to those which are marked in Supplementary Figure 1.

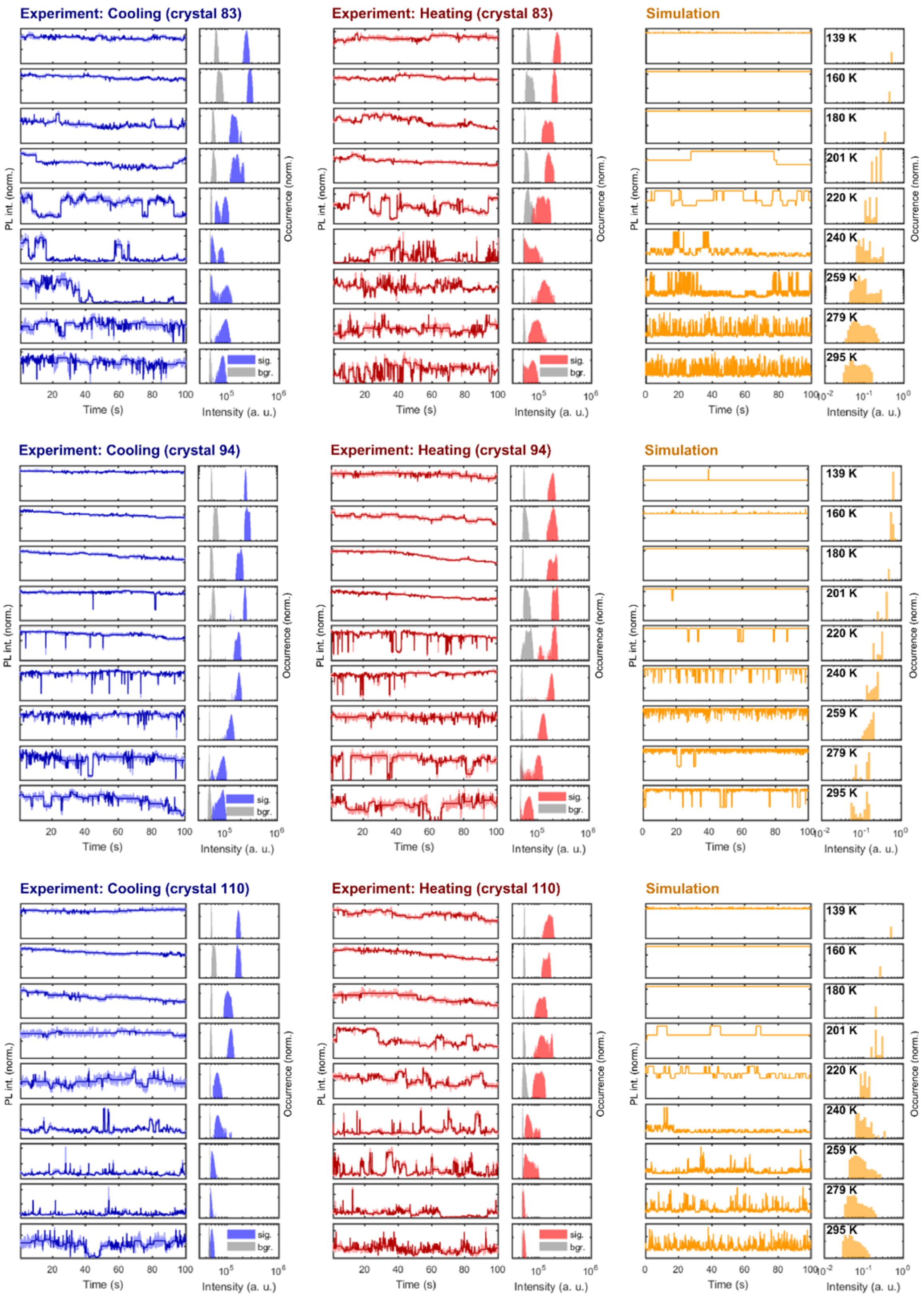

Supplementary Figure 6: Comparison between experiment and simulation for some crystals. Blue and red plots show experimental intensity transients and histograms for selected crystals at different temperatures. Yellow plots (right column) show simulated blinking transients with parameters selected to mimic the experimental data.

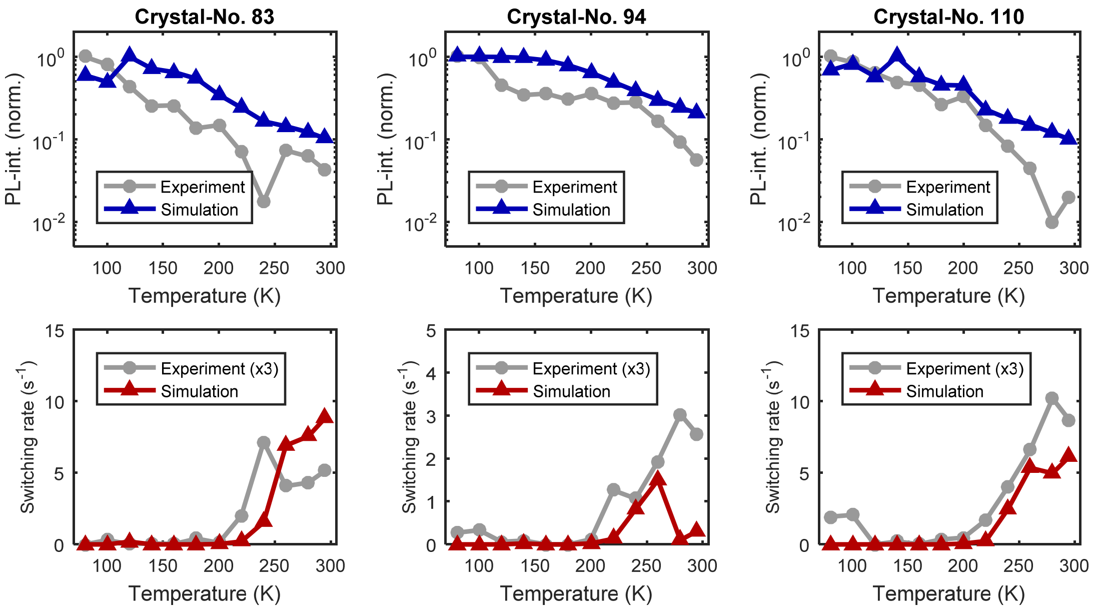

Supplementary Figure 7: Measured and simulated features extracted from the blinking transients. Simulated and experimental temperature dependencies of the PL intensity are shown in the upper row and the switching rates obtained from the data are presented in the lower row. The underlying blinking transients are shown in Supplementary Figure 6.

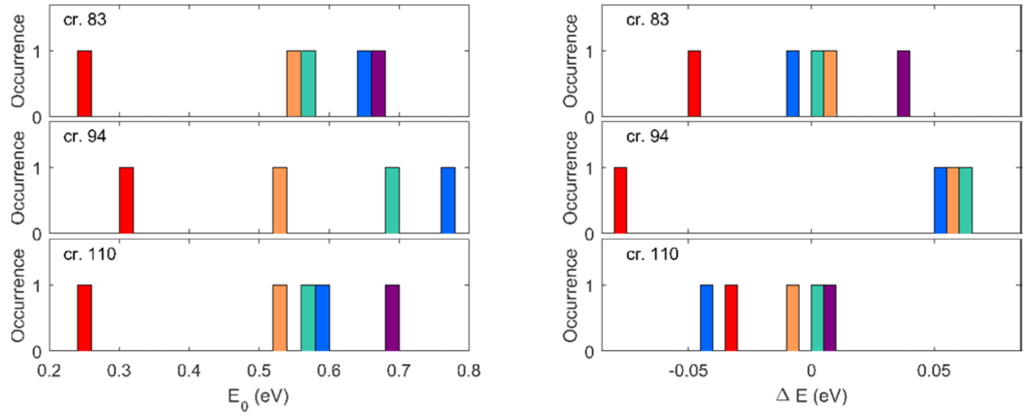

Supplementary Figure 8: Histogram-type illustration of the simulation parameters. The blinking transients resulting from this set of parameters are presented in Supplementary Figure 6. In all three examples, we used a number of four quenchers. The energy E_0 denotes the minimum barrier height the system has to be overcome to change the switching state. ΔE is the energetic difference between the active and the passive state (see Figure 5 in the main text).

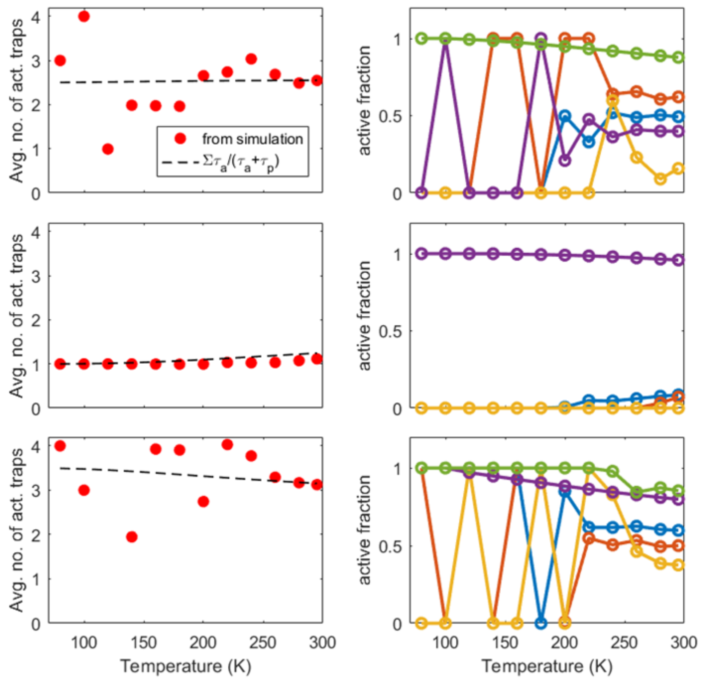

Supplementary Figure 9: Average number of active quenchers. The left column shows the average number of active traps obtained for the three simulated crystals (red dots). The values were calculated from the time-averaged sum over all random sequences $q_i(t)$, which describe the switching state of individual quenchers (see Equation 2 in the main text) plus the contribution of the fast switching quenchers, which is not contained in $Q_i(t)$ and instead given by the ratio of the characteristic switching times $\tau_{\text{active}}/(\tau_{\text{active}} + \tau_{\text{passive}})$. If all the quenchers are in thermal equilibrium (i.e. switching very fast compared to the time scale of the experiment), the number of active quenchers can be expressed by the sum over the ratio of switching times of all traps $\sum_i \tau_{\text{active},i}/(\tau_{\text{active},i} + \tau_{\text{passive},i})$, which is indicated by the dotted lines. At room temperature, more quenchers in our model are in equilibrium than at low temperature and thus, the number of active quenchers is closer to the calculated curve than at low temperature, where the switching times can be very long such that a quencher is either permanently active or passive. We assume that the 'freezing' of switching states at low temperature due to absence of thermal equilibrium is also responsible for the individual slopes observed in the temperature dependent PL intensity. In the right column we plot the contribution of individual traps to the time-averaged number of active traps. At low temperature, in most cases a 'digital' picture emerges, because the traps are either active or passive throughout the whole temperature range. At higher temperatures, several switching events between active and passive state occur and the fraction of active times takes values between 0 and 1.

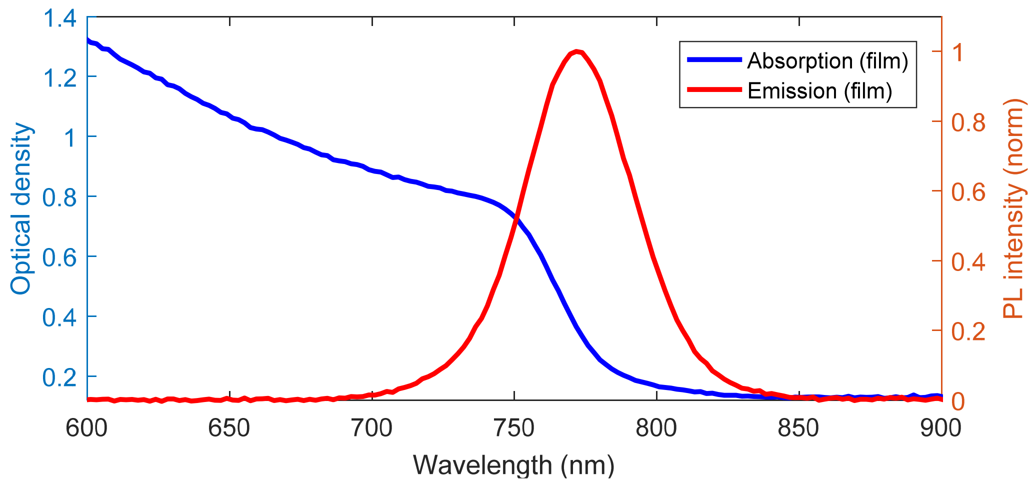

Supplementary Figure 10: Absorption and emission spectrum at room temperature. To measure absorption of the material, we produced a thin film of $MAPbI_3$ via the same method as for the nanocrystals, but with a higher concentration of the precursor solutions. The absorption spectrum (blue curve) was obtained using the same optical microscope as for detection of PL and imaging. We measured the spectrum of a white light lamp which was transmitted through the sample. Since the glass substrate was not fully covered with the material, both the reference and transmission spectra were recorded from the same sample (see details about the method published elsewhere).[1] The absorption edge of $MAPbI_3$ is well reproduced. Below the band edge, the absorption does not decline to zero, which we assume is due to scattering in the polycrystalline film.[1] In addition, we measured the PL emission spectrum at the same sample position (red curve). The PL spectrum of the film is red-shifted compared to the spectra of the nanocrystals (see Supplementary Figure 11). The origin of this effect is not fully clear to us, but we assume that reabsorption in the optically thick film could play an important role, rather than quantum size effects, because the crystals are much larger than the size typically associated with quantum confinement.

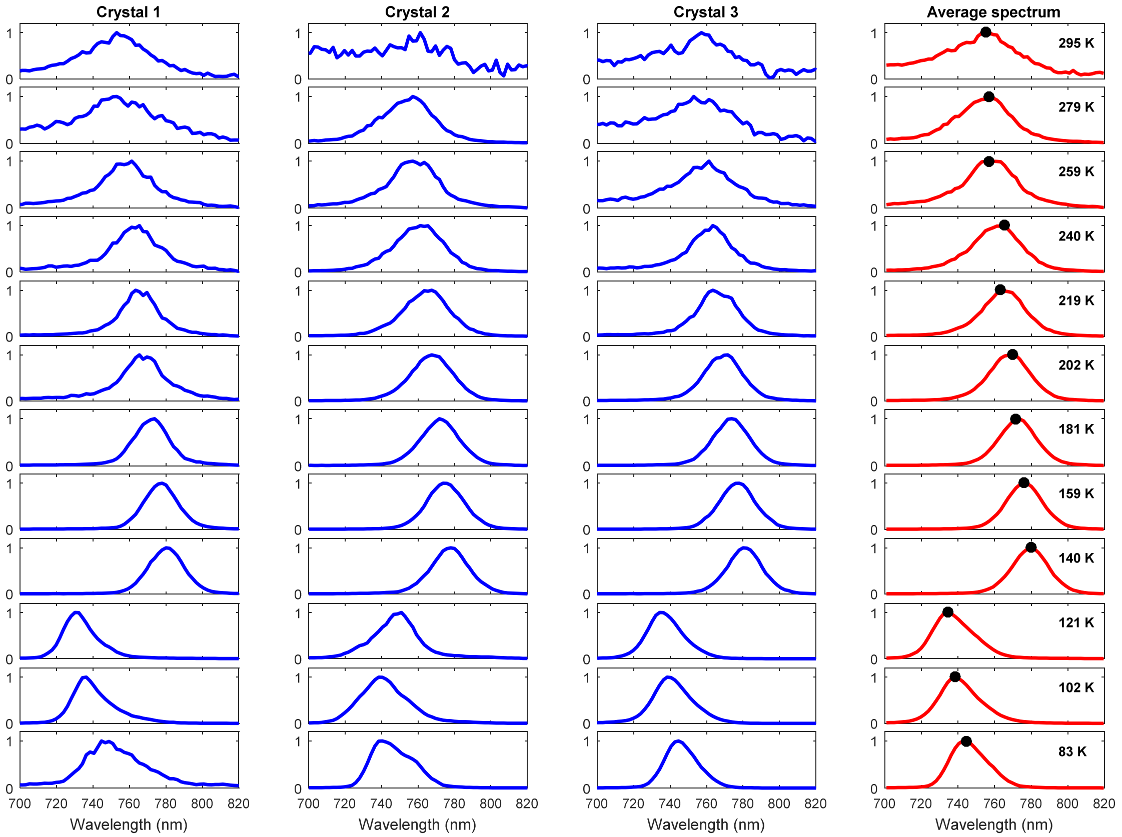

Supplementary Figure 11: Temperature dependent PL spectra. Here, we present data recorded for three different crystals (blue) and average spectra obtained from these curves (red, right column). The average spectra show the temperature-dependent position of the PL maximum. Upon cooling, we observe a red-shift of the emission maximum from approx. 760 nm at room temperature to 780 nm at 140 K. Between 140 and 120 K an abrupt blue shift of the emission maximum occurs, which is related to the phase transition from the tetragonal to the low-temperature orthorhombic phase with a higher energetic gap. The overall temperature dependence of the emission maximum is consistent with previous luminescence studies,[2] demonstrating the reliability of our low temperature setup.

Supplementary Note 1: Scanning electron microscopy and correlation with luminescence

Correlation with photoluminescence

Scanning electron microscopy (SEM) allows us to estimate the crystal size and provides insight into the morphology of the studied nanocrystals, which are smaller than the diffraction limit of the optical microscope. By overlapping the SEM data with the PL recorded from the same region, we find that, in most cases, the diffraction-limited spots contain only one single object. The SEM micrographs presented in the following reveal crystal diameters of approx. 70 nm. The best spatial resolution of the SEM is approx. 15 nm, thus it is difficult to determine, whether the crystals are comprised of several grains or whether it can be assumed that excitations can migrate freely within the whole crystal. A detailed view on some individual objects (insets in Supplementary Figure 12), however, suggests that pronounced grains are absent.

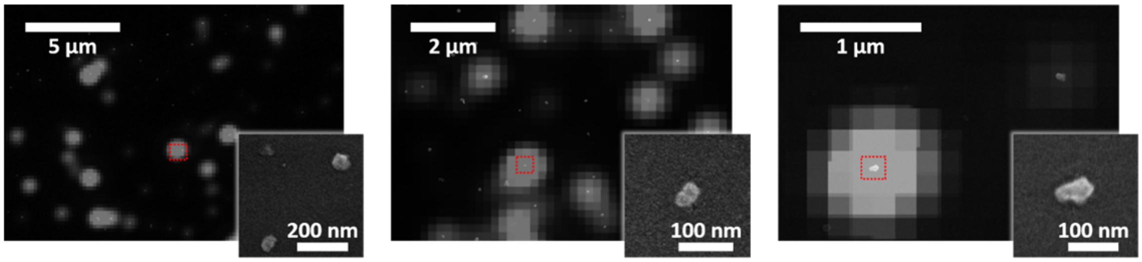

Supplementary Figure 12: Exemplary SEM data. The larger-scale maps show overlapped SEM and PL images. The PL images were acquired at a temperature of 80 K. Apparently, not all crystals detected with the electron microscope are also bright in luminescence. This indicates that even at low temperature a large fraction of the photoexcitations is subject to non-radiative losses in some crystals. The insets of the figures show exemplary high-resolution scans of individual crystals in the red-marked areas.

Estimation of the quenching volume

For some of the crystals, both blinking traces and SEM images are available. This data allows us to estimate the quenching volume. We use the micrographs to measure the sizes of the crystals in x and y direction, d_x and d_y, respectively. From this, we estimate the crystal volume via $V_\mathrm{c} = \pi \left(\frac{d}{2}\right)^2 \cdot d$, where $d = \frac{d_\mathrm{x}+d_\mathrm{y}}{2}$. The quenching volume is then estimated via $V_\mathrm{q} = A_\mathrm{max} \cdot V_\mathrm{c}$, where A_max is the maximum observed relative blinking amplitude.

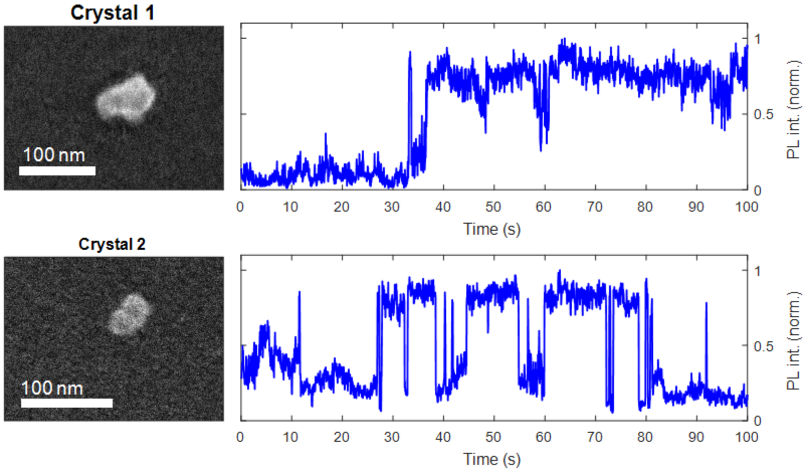

Supplementary Figure 13: SEM images (left) and blinking traces (right) recorded for two crystals.

	d_x (nm)	d_y (nm)	V_c (nm^3)	A_{max}	$\boldsymbol{V_q}$ **(nm^3)**
Crystal 1	58.6	82.9	2.8x10^5	0.63	**1.8x10^5**
Crystal 2	48.5	52.6	1.0x10^5	0.77	**7.8x10^4**

Supplementary Table 1: Estimation of the quenching volume. Quenching volumes V_q were exemplarily calculated for two crystals, for which we recorded blinking transients and SEM micrographs.

For both crystals, we find traps with quenching volumes on the order of 10^5 nm^3. However, this should be regarded as a lower boundary for the actual quenching volume, since we expect that the same quencher in a larger crystal could quench a much larger volume. This is due to the absence of saturation of the blinking amplitudes at a higher excitation power density (see Supplementary Note 4). In previous work, the quenching volume has been estimated from the amount of emitted photons and lead to a similar value of 7x10^4 nm^3.[3]

Size distribution of the Nanocrystals

In order to obtain some statistics of the crystal size, we recorded SEM images of larger regions. From these images, we estimated the crystal diameters employing a threshold-based image analysis routine. First, the routine detects all regions of bright pixels above a certain threshold, which correspond to individual crystals. Next, the horizontal and vertical diameters of these regions are measured. The histograms show the distributions of the extracted diameters. On average, we measure a crystal diameter of about 70 nm.

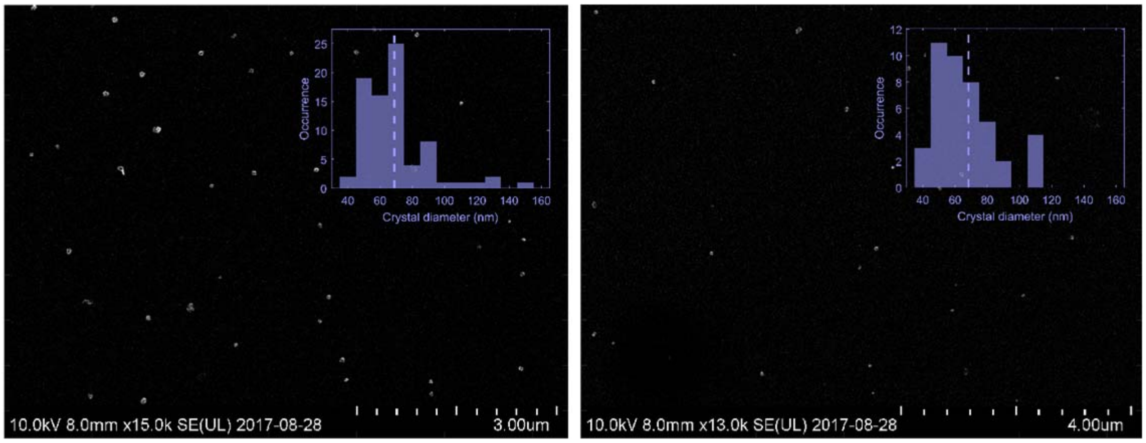

Supplementary Figure 14: Estimation of the crystal size. Here, two different sample regions were recorded by SEM. The insets show the distributions of the crystal diameters extracted from the respective images. The mean crystal diameter is approximately 70 nm, as indicated by the dashed lines in the histograms.

Supplementary Note 2: Estimation of the excitation density

In order to estimate the excitation density for individual crystals, we measured the integrated excitation power (10 µW for the temperature sweep) and recorded an image of the beam profile using fluorescent ink. Fitting the beam profile with a two-dimensional Gaussian function allowed us then to calculate the excitation power per area in every pixel of the image. Supplementary Figure 15(a) shows a map of the calculated excitation fluence, plotted together with the positions of the localized crystals. The histogram in Supplementary Figure 15(b) indicates, how many crystals were found in different ranges of excitation fluence. For continuous wave excitation at 458 nm, the ensemble-averaged excitation fluence is 0.3 W cm^{-2}, which is equivalent to an energy fluence of 3 suns.

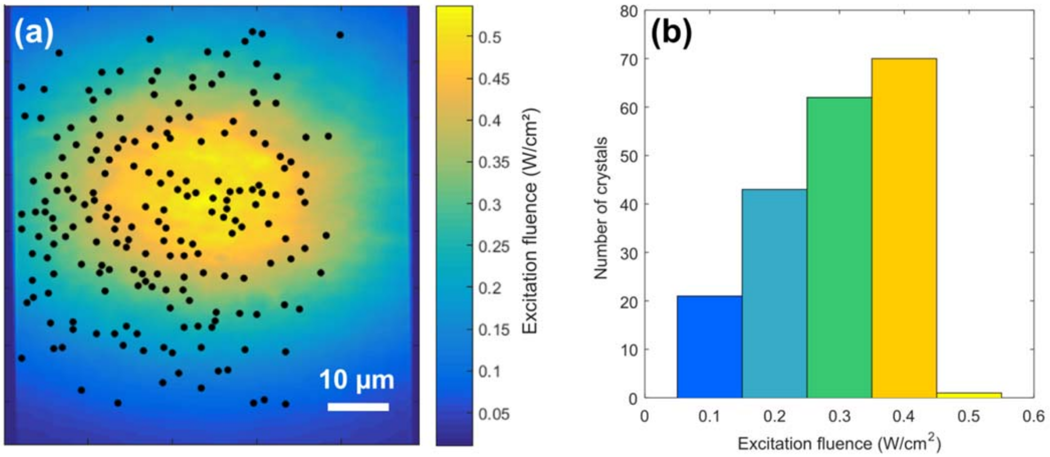

Supplementary Figure 15: Estimation of the excitation fluence. The map of the excitation fluence in (a) was calculated from a fit of the recorded beam profile and the measured integrated excitation power (10 µW). The beam diameter (full width at half maximum) is about 60 µm. The black dots indicate localized crystals within the excitation profile. With this information, we calculated the excitation fluence for each individual crystal. The distribution of excitation fluences is shown in (b).

In order to estimate the average number of excitations per crystal, the absorption coefficient of the material has to be taken into account. Here, we rely on literature values, since transmission measurements of diffraction-limited objects are difficult to realize and alternative approaches, like for example photoluminescence excitation spectroscopy, are not necessarily sensitive to the actual absorption of the sample, but rather show the concerted result of different phenomena. The absorption coefficients of $CH_3NH_3PbI_3$ reported in literature vary over a certain range, between approximately 1 x 10^{5} cm^{-1} and 3 x 10^{5} cm^{-1} at 458 nm.[4–7] Additionally, the absorption coefficient at 458 nm has been shown to increase by about 15 % when the material is cooled from room temperature to 77 K.[7] However, by applying Beer's law and considering a sample thickness of 70 nm, we estimate that the number of absorbed photons changes only by about 6 % and therefore neglect this effect.

The number of excitations created in one crystal per second is given by:

$$N = \frac{I_0 S}{h\nu}(1 - \exp(-\alpha d)) \qquad (1)$$

The photon energy at 458 nm is $h\nu = $ 4.34 x 10^{-19} J and the average excitation power density I_0 = 0.3 W cm^{-2}. Assuming a crystal thickness d = 70 nm (see Supplementary Note 1), an absorbing

surface $S = \pi \left(\frac{d}{2}\right)^2$ and an absorption coefficient $\alpha = 2 \times 10^5$ cm^{-1},[5] we estimate an average number of 2×10^7 s^{-1} excitations per crystal.

Considering an average excitation power density of 0.3 W cm^{-2} and a carrier lifetime τ below 50 ns, we calculate a number of $N_0 = N \cdot \tau < 1$ excitations per crystal. However, this value is expected to vary among the ensemble of crystals due to their size and lifetime distribution and a position-dependent excitation fluence. Taking these effects into account, as well as a limited accuracy of the absorption coefficient, variations between 0.05 and 5 excitations per crystal appear realistic. This corresponds to an average excitation density of 3.7×10^{15} cm^{-3}.

Assuming that the recombination constants of our $MAPbI_3$ crystals are similar to other reports ($k_1 = 10^6$ to 10^8 s^{-1}, $k_2 = 10^{-3}$ cm^3s^{-1} and $k_3 = 10^{-28}$ cm^6s^{-1}),[8] we infer that the regime of excitation densities employed in our study is far below the onset of Auger recombination. A detailed analysis of the dominant recombination mechanism as function of excitation power has been carried out by Johnston and Herz.[8] They show that for realistic monomolecular decay rates, Auger recombination starts to dominate the decay for charge carrier densities above 10^{17} cm^{-3}. The absence of substantial Auger decay under the experimental conditions applied in this work is further supported by our experimental data, which shows an increase of the PL quantum yield with increasing excitation intensity (see Supplementary Figure 17).

Supplementary Note 3: Estimation of the photoluminescence quantum yield (PLQY)

As has been shown for individual molecules, having a properly calibrated fluorescence microscope allows for quantitative measurement of fluorescence quantum yield of nano-sized emitters if their absorption cross section is known.[9] Our microscope is the same as was used before for the mentioned single molecule brightness measurements and its calibration data is available.[9] Therefore, in order to calculate quantum yield we need to find perovskite crystals for which we have SEM images (to estimate absorption cross section from their physical size) and PL intensity transients. Such crystals are those shown in Supplementary Figure 13. From the crystal size (see Supplementary Table 1) and the excitation power density we can estimate the PL quantum yield (PLQY) of the studied crystals from the following equation:

$$PLQY = \frac{N_{\mathrm{em}}}{N_{\mathrm{abs}}} = \frac{I_{\mathrm{CCD}} \cdot f_{\mathrm{setup}}}{PD \cdot S \cdot (1 - \exp(-\alpha \cdot d)) \cdot \frac{\lambda}{hc}} \tag{2}$$

where N_{em} is the number of emitted photons, which is calculated from the number of counts I_{CCD} detected by the CCD camera and a setup specific factor f_{setup}, which is 13.1 in our case. The number of absorbed photons N_{abs} is calculated from the excitation power density PD at the crystal location, the crystal diameter d, the crystal cross section $S = \pi \left(\frac{d}{2}\right)^2$, the absorption coefficient α = 2 x 10^7 m^{-1} and the inverse of the photon energy $\frac{\lambda}{hc}$ corresponding to the excitation wavelength λ = 458 nm.

At room temperature, the estimated PLQY of crystal 1 is 0.2 % and the yield of crystal 2 is 1.9 %. These numbers are in accordance with the observed increase of the PL intensity upon cooling. Since the ensemble emission at 80 K was about two orders of magnitude higher than at room temperature, we would not expect calculated room temperature quantum yields significantly above 1 %. At lower temperatures, the PLQY reaches values around 20 %. This indicates that even at temperatures as low as 80 K some of the non-radiative channels remain active and lead to efficient quenching of the PL (the pre-factor $\Phi_0(T)$ in Equation 2 in the main text).

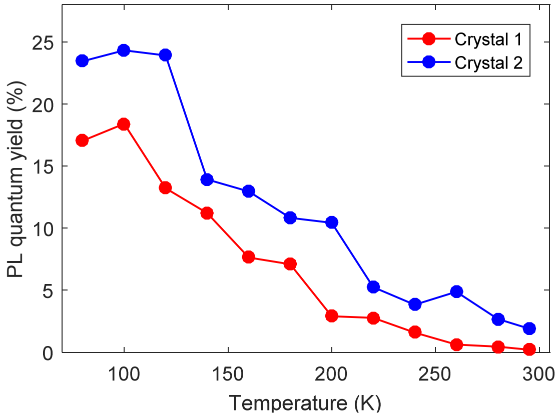

Supplementary Figure 16: Estimated PLQY at different temperatures. Blinking transients and SEM images of the crystals are shown in Supplementary Figure 13.

Supplementary Note 4: Excitation power dependent measurements

By varying the excitation power density over two orders of magnitude, we observe a super-linear increase of the PL intensity (Supplementary Figure 17), which can be fit with a power-law function, yielding exponents larger than one. This increase of the PL quantum yield with increasing excitation power is in accordance with previous measurements at low temperature[10–12] and results from the power-dependent competition of radiative and non-radiative channels.

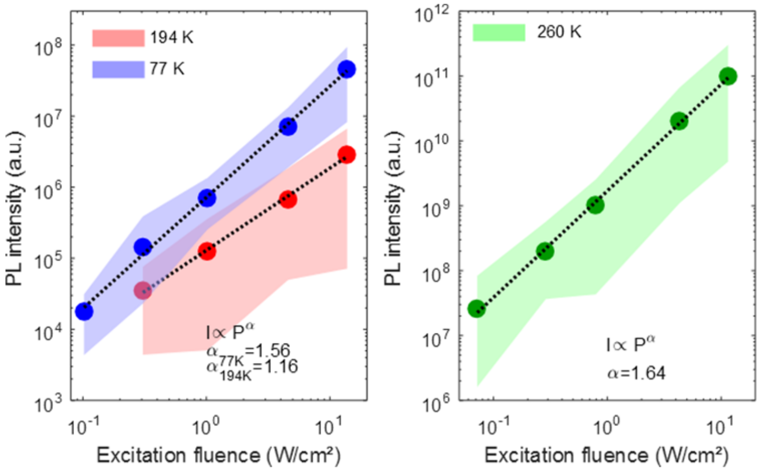

Supplementary Figure 17: PL intensity as function of ensemble-averaged excitation fluence. The power-law fits with exponents larger than 1 demonstrate a superlinear increase of the PL intensity. The markers indicate the mean intensity and the shaded areas correspond to the intensity range containing 80 % of the data. The data at 77 and 194 K (left panel) was recorded from the same sample that served also for the temperature sweep presented in the main text. The data at 260 K (right panel) was recorded from a later batch that was prepared with the same method.

Saturation effects

Our analysis of power-dependent blinking amplitudes suggests that blinking is rather insensitive to the excitation power density. Complementary CDF plots of the extracted intensity jumps are presented in Supplementary Figure 18. The slopes of the CDF plots are similar, suggesting that with increasing excitation power, the blinking amplitudes increase in a similar way as the PL intensity. We also note the shoulders appearing in the CDF plots for the highest excitation power density, which signify an increasing probability of observing large blink events. The origin of this peculiar change of the amplitude distribution at high excitation power density is not yet clear to us. However, we can conclude that a higher excitation power density does not lead to any clear decrease of the quenching ability of the fluctuating quenchers. Thus, a trap filling effect (saturation) for the fluctuating non-radiative channel can be ignored at first approximation when an excitation power density of a few suns is used.

Contrary to our results, in the study of Tian et al.[3] a reduction of the blinking amplitudes with increasing excitation power has been clearly observed. Here, it is important to note that the crystals in their work were much larger in size, such that they could access a much higher number of photoexcitations, leading to trap saturation in case of high excitation power densities.

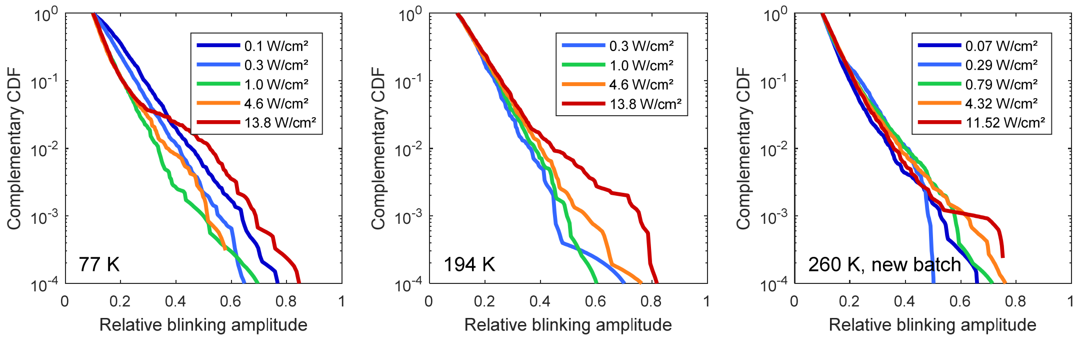

Supplementary Figure 18: Power- and temperature dependent CDF plots of relative blinking amplitudes. Here, we considered only intensity jumps with amplitudes exceeding 10 % of maximum intensity. The data suggests that there is no remarkable influence of the excitation power density on the blinking amplitudes, i.e. no saturation of the fluctuating quenchers is observed.

Photoactivation of blinking

In the following, we investigate if the blinking mechanism is photo-activated. Since the recorded blinking transients reveal complicated switching behavior, we do not perform a conventional analysis of the ON and OFF times. Instead, we plot the switching rate as function of excitation power density. The left panel of Supplementary Figure 19 shows the switching rate averaged over an ensemble of 45 crystals. In addition, we analyze the probability of the crystals to reside at a particular intensity level (right panel of Supplementary Figure 19), which has been calculated from the normalized intensity histograms at a given excitation power density. As a result, we do not observe a clear dependence of the blinking behavior on excitation power density.

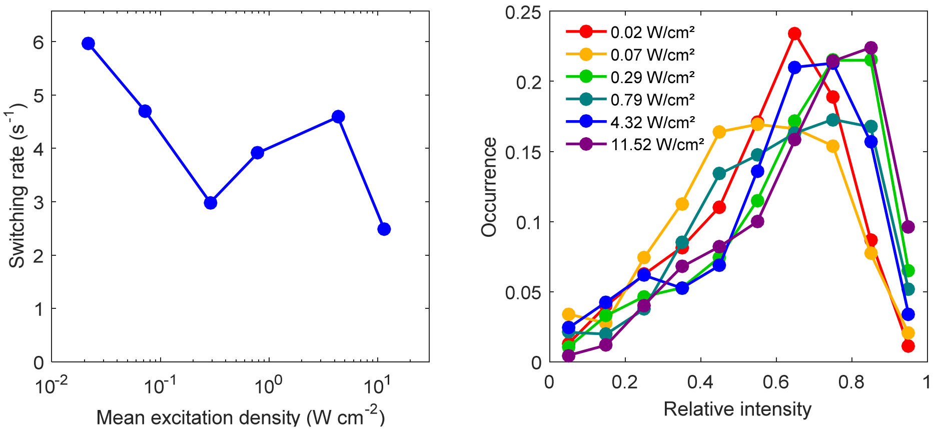

Supplementary Figure 19: Switching behavior as function of excitation density recorded at 260 K. Here, all intensity jumps exceeding 10 % of the maximum intensity were considered. The plot on the left shows the mean switching rate of an ensemble of 45 crystals. The right panel shows the ensemble-averaged probability to find the crystal at a specific intensity level normalized to one. No clear correlation with the excitation power density can be found.

Since we speculate that the blinking mechanism observed in our work is related to ion migration, which is known to be photo-activated, the absence of photoactivation effects in the switching behavior is a bit surprising. The reason for this could be that the regime of excitation densities we probe is actually too high to see the onset of photo-activation that would lead to increased blinking activity. A study on photo-activated ion migration reports significant changes of the ionic conductivity for excitation powers below 0.02 W cm^{-2} with a tendency to saturate at higher excitation powers.[13] In our experiment, however, we applied excitation densities ranging between 0.02 W cm^{-2} and 12 W cm^{-2}. We therefore speculate that the effect of photoactivation plays only a minor role.

Supplementary note 5: Time-resolved photoluminescence (TRPL)

TRPL at room temperature

In the following, we provide an overview of the PL dynamics of the investigated nanocrystals. For lifetime analysis, the data can be fit with a triexponential function:

$$PL(t) = I_0 + A_1 \exp\left(-\frac{t}{\tau_1}\right) + A_2 \exp\left(-\frac{t}{\tau_2}\right) + A_3 \exp\left(-\frac{t}{\tau_3}\right) \quad (3)$$

Note that the choice of the fit function is not related to any specific physical model, but it allows us to measure the decay dynamics by calculating the amplitude averaged lifetime $\langle\tau\rangle$ from the fit parameters:

$$\langle\tau\rangle = \frac{A_1\tau_1 + A_2\tau_2 + A_3\tau_3}{A_1 + A_2 + A_3} \quad (4)$$

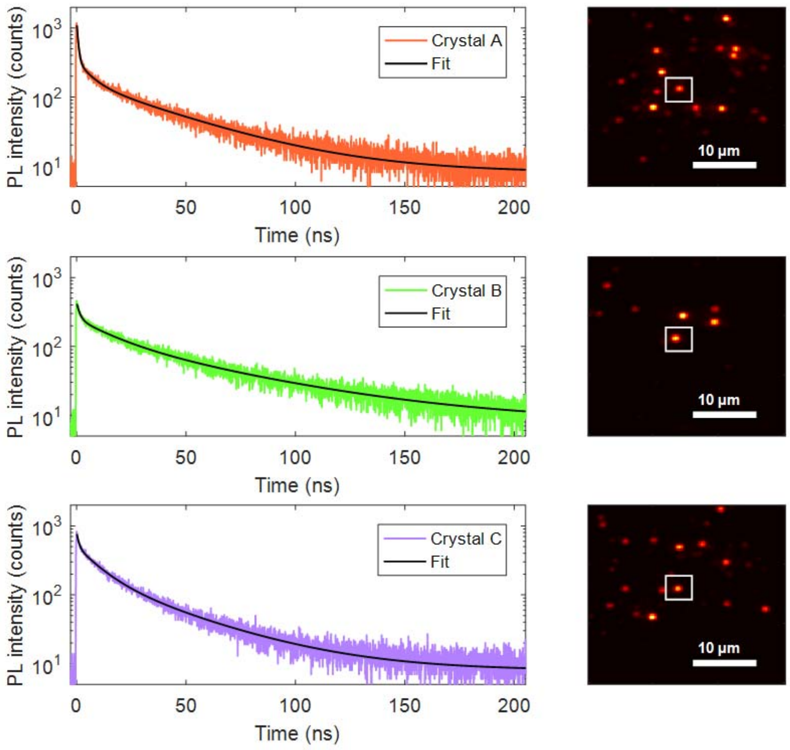

Supplementary Figure 20: Room temperature PL dynamics. We investigated different crystals in the sample region, which was also used for the temperature dependent blinking measurements. For excitation, we used a pulsed diode laser operating at 485 nm and a repetition rate of 2.5 MHz. We estimate the excitation energy per pulse to 0.13 µJ cm^{-2}, from which we calculated a carrier density of 5 x 10^{16} cm^{-3}, considering a crystal thickness of 50 nm and an absorption coefficient of $MAPbI_3$ of 2 x 10^5 cm^{-1}. The spectrally integrated signal was recorded with a time-correlated single photon counting system (PicoQuant).

	A_1	A_2	A_3	τ_1 (ns)	τ_2 (ns)	τ_3 (ns)	$\langle\tau\rangle$ **(ns)**
Crystal A	908 ± 6	175 ± 4	160 ± 2	0.69 ± 0.01	6.08 ± 0.21	38.38 ± 0.39	**6.30**
Crystal B	176 ± 4	127 ± 4	120 ± 5	1.44 ± 0.06	15.76 ± 0.76	57.59 ± 1.24	**21.62**
Crystal C	316 ± 5	298 ± 5	182 ± 6	1.00 ± 0.03	9.89 ± 0.25	36.02 ± 0.62	**12.53**

Supplementary Table 2: Fitting results. Amplitudes and lifetimes of the three contributions to tri-exponential decay were obtained from fitting Equation 3 to the transients in Supplementary Figure 20.

Temperature dependent TRPL

To measure the PL decay as function of temperature, the sample was prepared following the same procedure as for the sample that served for the measurements presented in the manuscript. Transients at different temperatures were recorded with a photon counting system for five different crystals. Exemplary transients recorded for one of these crystals are presented in Supplementary Figure 21(a). The temperature dependent amplitude averaged lifetimes $\langle\tau\rangle$ are summarized in Supplementary Figure 21(b). Upon cooling, we first observe an increase of the effective lifetimes, followed by a decrease below ca. 180 K. This behavior could be due to the change of the dominating recombination mechanism from monomolecular to bimolecular, because the decreasing decay times with decreasing temperature are accompanied by a rise of the overall PL intensity.

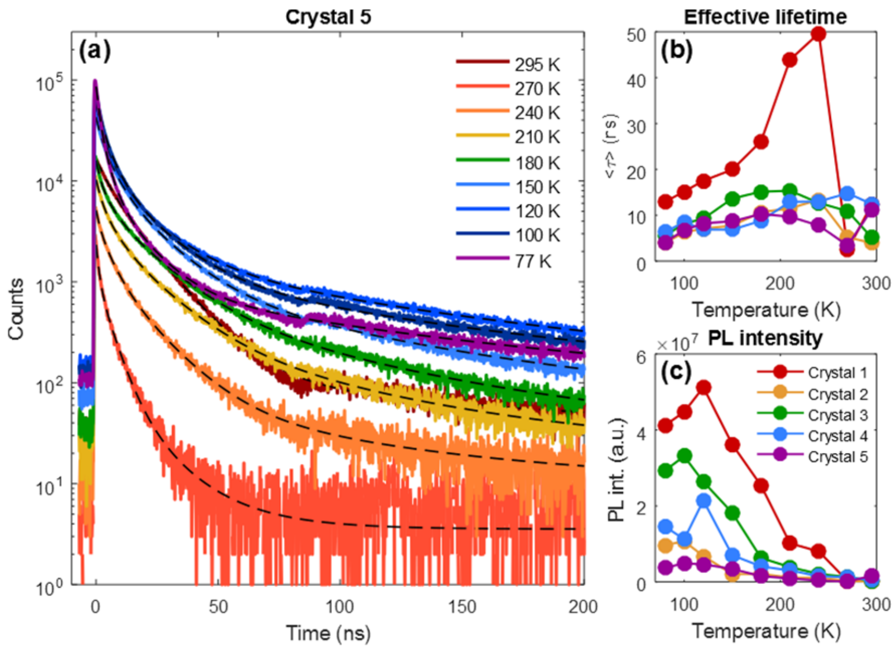

Supplementary Figure 21: PL decay transients at different temperatures. The data was recorded with a photon counting system (PicoQuant), including a pulsed diode laser operating at 485 nm and an APD detector. For each measurement, an integration time of 100 s was used. For the five crystals under investigation, we calculated excitation power densities between 0.2 and 0.6 W cm^{-2}, depending on their position in the excitation spot. With the repetition rate of 2.5 MHz, this corresponds to pulse energies of 0.8 to 0.24 µJ cm^{-2}. We recorded temperature dependent decay curves for a group of 5 crystals. The data was fit with a triexponential function, as demonstrated by the dashed lines in the left panel. From the fit parameters, we calculated the amplitude averaged lifetime $\langle\tau\rangle$, which is plotted in panel (b). Panel (c) shows the integrated PL intensity that was obtained from the recorded decay curves.

Supplementary Note 6: Estimation of the fraction of free charges

Photoexcitation leads to co-existence two excited species in semiconductors, free carriers and excitons, which are in dynamic equilibrium. The equilibrium depends on the carrier density and exciton binding energy and can be expressed by the Saha-Langmuir-equation:

$$\frac{n^2}{1-n} = \left(\frac{2\pi\mu k_\mathrm{B}T}{h^2}\right)\cdot\exp\left(-\frac{E_\mathrm{B}}{k_\mathrm{B}T}\right) \tag{5}$$

Here, n denotes the concentration of free carriers (either electrons or holes), μ is the reduced mass of the exciton (here: μ = 0.1 m_e),[14] k_B the Boltzmann-constant, T the temperature, h is Planck's constant and E_B the exciton binding energy. The right side of the equation can be regarded as an equilibrium constant $c(T)$. Note that we can express the overall fraction of charges as $\xi_\mathrm{c} = n/N$, where N is the overall density of photoexcitations. Rewriting the equation above yields:

$$\frac{\xi_\mathrm{c}^2}{1-\xi_\mathrm{c}} = \frac{c(T)}{N} \tag{6}$$

Solving the quadratic equation leads to:

$$\xi_\mathrm{c}(T) = \frac{c(T)}{2N}\left(\sqrt{1+\frac{4N}{c(T)}}-1\right) \tag{7}$$

Due to the high diversity of literature values reported for the exciton binding energy, we plot $\xi_\mathrm{x}(T)$ for a broad range of E_B between 5 and 60 meV. However, binding energies below 25 meV appear more realistic,[14,15] since higher values were often obtained from fitting the temperature dependent PL intensity, which we do not consider as a reliable method, as discussed in the manuscript. Moreover, following our estimate of the excitation density (N = 4 x 10^{15} cm^{-3}, see Supplementary Note 2), we explore a range of excitation densities between N = 4 x 10^{14} cm^{-3} and 4 x 10^{16} cm^{-3}.

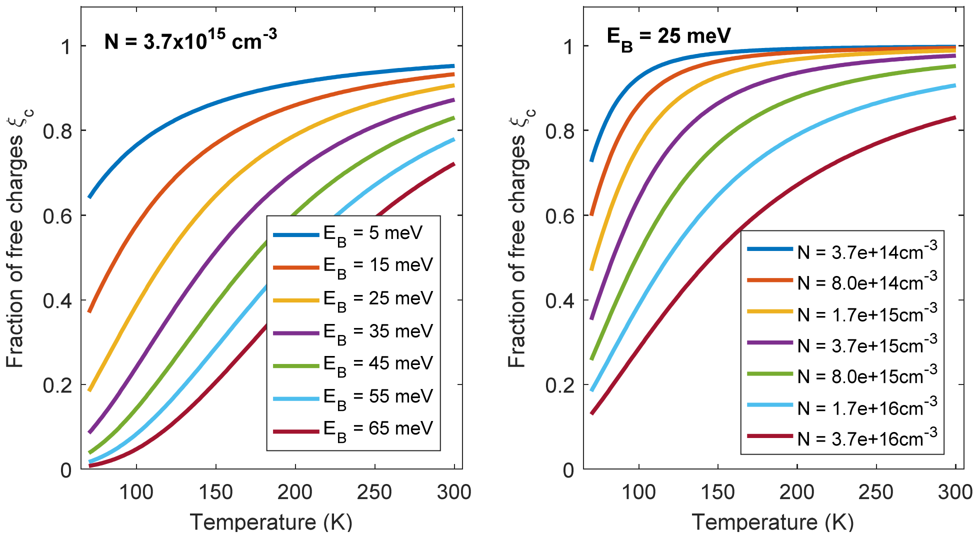

Supplementary Figure 22: Estimation of the fraction of free charges. Calculations were carried out for different exciton binding energies (left) and excitation densities (right).

It turns out that at room temperature, the fraction of excitons is small, most likely below 10 %. At temperatures as low as 80 K ($k_\mathrm{B}T$ = 7 meV), however, an excitonic fraction on the order of 50 % is not unrealistic. It is thus important to discuss whether the traps that we study in this work actually reveal similar capture cross sections for both photoexcited species.

A key observation we made in our experiments was the decrease of the blinking amplitudes with decreasing temperature, which indicates a reduction of the quenching efficiency of the non-radiative centers. A potential explanation for this behavior could be different capture cross sections of the non-radiative centers for free carriers and for excitons, such that the higher excitonic population at lower temperatures would impede high quenching efficiencies. The plots in Supplementary Figure 22 reveal that one would indeed expect a higher fraction of excitons at low temperature, however, we believe that the changing excitonic population is not responsible for the observed temperature dependence of the blinking amplitudes. This is mainly because in the experiments we observed a decrease of the blinking amplitudes by about one order of magnitude upon cooling the samples from room temperature to 80 K. However, according to Supplementary Figure 22, the expected change of the fraction of free carriers is less strong. Furthermore, we note that excitons and charges are in dynamic equilibrium, which means that during their lifetime, excitons can split into free charges and form the bound state for many times. Thus, even though the quenchers are more selective to charges, excitons could be quenched during the time they are dissociated. Note that pronounced blinking has also been reported for bromide perovskites ($MAPbBr_3$),[16,17] where the exciton binding energy is remarkably higher than in $MAPbI_3$.

Supplementary Note 7: Correlation between PL intensities and PL lifetime

In order to investigate the quenching mechanism in greater detail, we recorded blinking transients with an APD, employing time-tagged single photon counting. This technique allowed us to analyze the PL dynamics corresponding to different intensity ranges of a blinking transient. Supplementary Figure 23 shows blinking transients (first and third row) and decay curves associated with different intensity ranges of these blinking transients (second and fourth row). Here, we divided the blinking transients into two equally-sized intensity ranges marked with red and blue color. The PL decay transients represent the mean decay curves associated with these intensity ranges and demonstrate different decay dynamics.

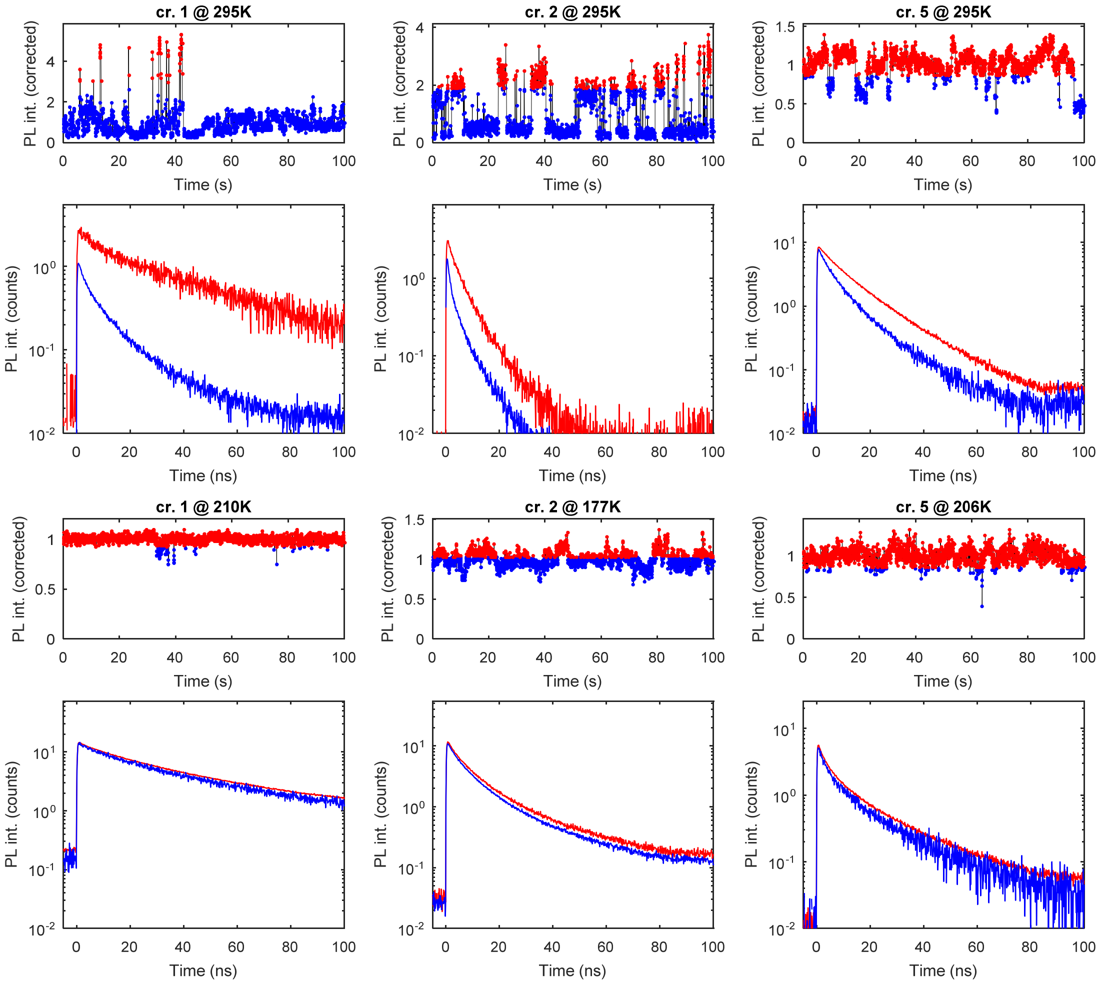

Supplementary Figure 23: Connection between PL dynamics and PL intensity in the blinking transients. Here, blinking transients were recorded for three crystals at different temperatures as labelled (see first and third row). In the transients, we set a threshold to subdivide them into bright and dim subsets. Red color denotes the bright subset and blue color is used to mark the dim subset. Decay curves associated with the different intensity intervals are presented in the second and fourth row. The overall intensity of the decay curves corresponds to the mean intensity in the respective intensity intervals.

To analyze the interrelation between PL intensity and PL dynamics in greater detail, it is helpful to evaluate the PL decay in smaller segments of the intensity transients. For the correlation of the PL intensity with the PL lifetime, we distinguish two limiting cases (see Supplementary Figure 24). In the first case, the intensity decays from a brighter level (I_1) to a dimmer level (I_2) without a noticeable change of the lifetime (static or ultrafast quenching). Such behavior can be associated with a

situation where only a small fraction of the excitations in a certain volume around a trap can be accessed by this quencher (fast process), whereas the majority of the excitations does not follow this decay channel and thus reveals similar decay behavior as in the bright state. Static quenching is thus a strong indicator for a diffusion-limited quenching process. In the second case, the PL lifetime is proportional to the PL intensity, i.e. a lower intensity level is also associated with faster decay. In this case, radiative and non-radiative channels have access to the same pool of photoexcited states and the non-radiative rate is limited by the capture cross-section of the quencher.

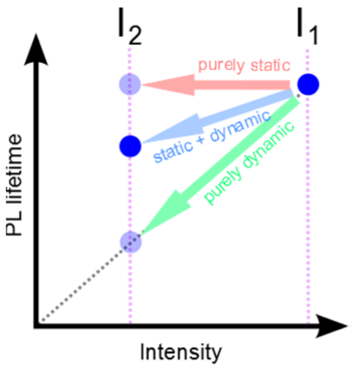

Supplementary Figure 24: Illustration of different quenching mechanisms. Both processes illustrated here can be related to a decay of the PL intensity from an initial value I_1 to a lower value I_2. If the PL intensity changes without a change of the PL lifetime (red arrow), the quenching process is limited by the diffusion of photoexcitations towards the trap. Since only a small volume in the vicinity of the trap is quenched and the decay dynamics in most of the regions of the crystal are not affected, the process appears as static. If, on the other hand, the lifetime is proportional to the PL intensity (green arrow), the non-radiative decay through the quencher is limited by the capture cross section, whereas diffusion does not set a limitation. This process becomes apparent as dynamic quenching. In reality, we observe a combination of both scenarios (blue arrow), i.e. the lifetime depends on the emission intensity but this dependence is not as strong as would be expected for purely dynamic quenching in a cross-section limited process.

To quantify the PL dynamics associated with the bright and the dim intensity ranges, we determine the amplitude-averaged PL lifetimes, which are generally defined as

$$\langle\tau\rangle = \frac{1}{I_0}\int PL(t)dt \tag{8}$$

Here, I_0 denotes the initial amplitude of the transient. Detailed discussion of this equation is given elsewhere.[18] In case of our measurements, the integral turns into a sum over all decay components and their amplitudes. The amplitude averaged lifetime can either be calculated from the parameters of a multiexponential fit, as demonstrated in Supplementary Note 5, or it can be calculated from the sum over all time bins with a bin size Δt = 2 ns in our case. The advantage of the latter method is that it is free of fitting and allows to process a big amount of data in a short time. Thus it was employed in the following.

In Supplementary Figure 25, we plot the lifetimes $\langle\tau\rangle$ as function of the normalized PL intensity at room temperature and at lower temperatures (175 – 210 K). So far, we do not attempt to give a quantitative picture about the contributions of static and dynamic quenching at different temperatures, since our dataset of blinking transients, especially at lower temperatures, is very limited. In addition, there might be inaccuracies of initial amplitude and the PL intensity, which should be taken into account when the data is interpreted.

Despite these issues, we observe a clear dependence of the PL lifetime on emission intensity. In most cases, this dependence is weaker than what would be expected for exclusively cross-section limited quenching, i.e. when the linear slope is extrapolated to zero intensity, the lifetime does not become

zero. From this we conclude that there must be a subset of excitations which cannot reach the quenchers via diffusion. This finding is not surprising to us, because in bigger crystals, diffusion-limited quenching has been observed even at room temperature.[19] We speculate that beside the crystal size, also other parameters like the presence of internal grains and defects play an important role and can lead to differences in the presence of diffusion-limited quenching in different crystals. Further investigations of this diffusion-limited subset and its temperature dependence will certainly be part of future work.

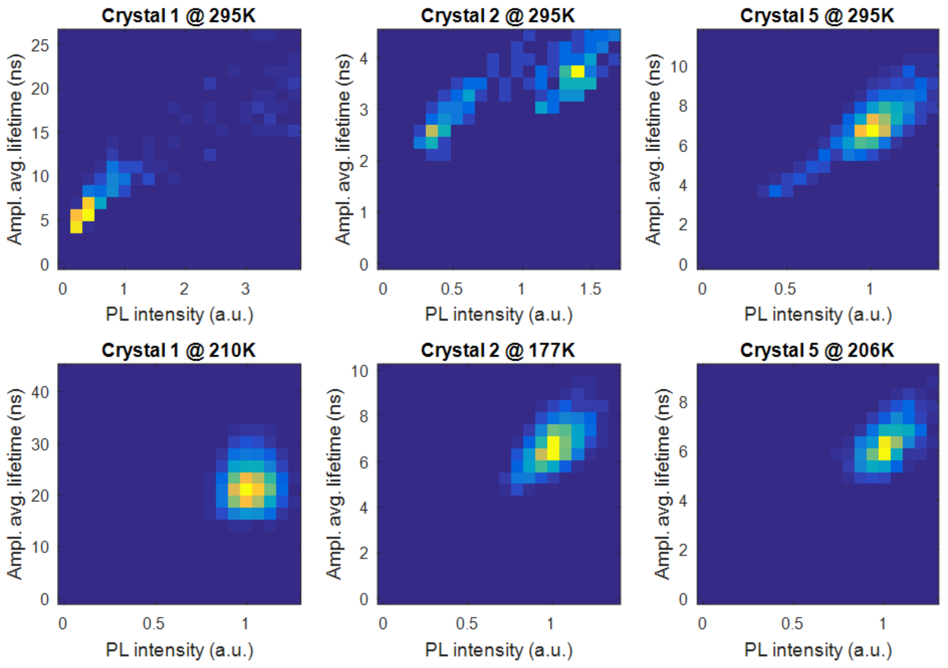

Supplementary Figure 25: Lifetime intensity correlation plots. The plots presented here are 2D histograms of the actual lifetime vs. intensity data and a brighter color indicates a higher density of points. Plots of the effective lifetime versus PL intensity at room temperature are presented in the upper row and correlation plots in the temperature range between 175 and 210 K are shown in the lower row for several individual crystals. Lifetimes were obtained by calculating the amplitude-averaged lifetime of a number of 1000 subsequent counted events (photons) that were binned with a time resolution of 2 ns. The corresponding PL intensity is then proportional to the inverse of the time, during which these 1000 photons are counted.

Supplementary Method 1: Selection of crystals for blinking analysis

Not all the crystals in the recorded were considered suitable for further analysis, for example because of an insufficient signal-to-noise ratio or because they are too close to other crystals. To address these issues, we included several selection steps in the data extraction procedure.

Localization of single crystals. Crystals were localized by finding local maxima in the drift-corrected and time-averaged PL movies above a certain threshold. We performed the localization procedure on data recorded at low temperatures, where more crystals could be detected. Crystals in too close proximity to each other were excluded from further analysis at this stage. Intensity transients of the signal and the background were then extracted from all drift-corrected movies at the same localization coordinates.

Signal-to-noise-ratio (SNR). The SNR of the time-averaged PL signal is defined via:

$$SNR = \frac{\langle I_{\mathrm{sg}} \rangle - \langle I_{\mathrm{bg}} \rangle}{std\left(I_{\mathrm{bg}}\right)} \tag{9}$$

Here, I_{sg} denotes the recorded intensity transient, I_{bg} is the intensity transient of the background recorded from a region nearby, the brackets $\langle * \rangle$ signify the time average and $std(I_{\mathrm{bg}})$ is the standard deviation of the background intensity transient. In order to contribute to the data analysis, we required a minimum SNR of 2. The number of crystals fulfilling this criterion is presented in Supplementary Figure 26. The decrease of the SNR with increasing temperature leads to the situation that many of the crystals cannot be detected at elevated temperatures. Thus, it is important to consider that the crystals being observed at room temperature actually represent a subset of the whole group with high quantum yield.

Two-dimensional Gaussian fitting. We assume that the crystal sizes are well below the diffraction limit of the optical microscope (see electron microscopy images in Supplementary Note 1), therefore the intensity profiles of the crystals should appear as diffraction-limited spots. Here, we fit the time-averaged intensity profiles of the crystals with a two-dimensional Gaussian function, which approximately corresponds to the point spread function (PSF) of the setup. When one of the fitting parameters for a crystal derived more from the PSF than a pre-defined tolerance, it was excluded from further analysis.

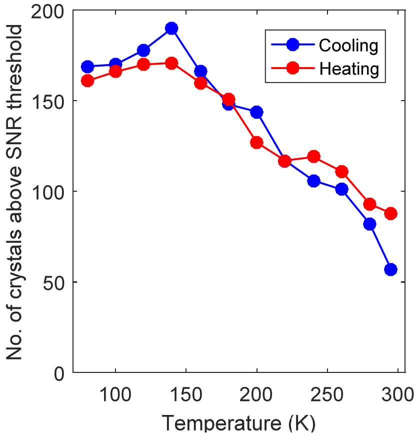

Supplementary Figure 26: Number of crystals above a pre-defined SNR of 2.

Supplementary Method 2: Simulation details

Temperature dependent ratio between radiative and non-radiative recombination

The experimental observation that the PL blinking amplitudes reduce upon cooling together with an overall increase of the PL intensity indicates that radiative recombination becomes more efficient compared to non-radiative recombination at low temperatures. Although we cannot assess the temperature dependencies of the individual radiative and non-radiative rates, analysis of the blinking amplitudes allows us to estimate the ratio $\kappa(T)$ between the fluctuating and non-fluctuating recombination rates as function of temperature. This function serves as important input parameter in our temperature dependent modeling (see Equation 2 in the main text).

We estimate $\kappa(T)$ from the lowest intensity levels extracted from the normalized blinking transients. Taking the lowest intensity levels of each transient might lead to overestimation of the overall non-radiative rate, however, we argue that these levels are least likely affected by time-averaging effects and thus most reliable to represent the quenching ability of one particular quencher. Note that even if two or more quenchers were active at the same time, the intensity level would still largely correspond to the effect of one quencher. Imagine e.g. a system with two quenchers, each of them with a quenching efficiency of 90 %. The first quencher reduces the PL intensity to 10 %, but if the second quencher is activated at the same time, it can only quench a much smaller fraction of the overall PL intensity (due to competing with the first quencher), so its influence is naturally smaller.

Supplementary Figure 27 shows the mean lowest intensity levels as function of temperature. The values were calculated from the distributions presented in Figure 3 in the main text. Due to the variety of influences on the radiative and non-radiative rate, we do not know the functional interrelation between the lowest intensity levels and temperature. However, we find that the slope of the data in Supplementary Figure 27(a) can be reproduced by fitting an activation law (see dashed line):

$$I_{\mathrm{rel}}(T) = \frac{I_0}{1 + a \cdot \exp\left(-\frac{E_{\mathrm{a}}}{k_{\mathrm{B}}T}\right)} \tag{10}$$

The fit yields the following parameters:

Parameter	Value
I_0	0.66 ± 0.02
a	421 ± 320
E_{a}[eV]	0.11 ± 0.02

Supplementary Table 3: Parameters obtained from activation law fit.

Since the lowest intensity levels presented here were extracted from the normalized blinking transients, they are directly related to the ratio of the radiative and non-radiative rate $\kappa(T)$:

$$I_{\mathrm{rel}}(T) = \frac{1}{1 + \kappa(T)} \tag{11}$$

From this expression and with the experimental input $I_{\mathrm{rel}}(T)$, we calculate $\kappa(T)$, as presented in Supplementary Figure 27 (b).

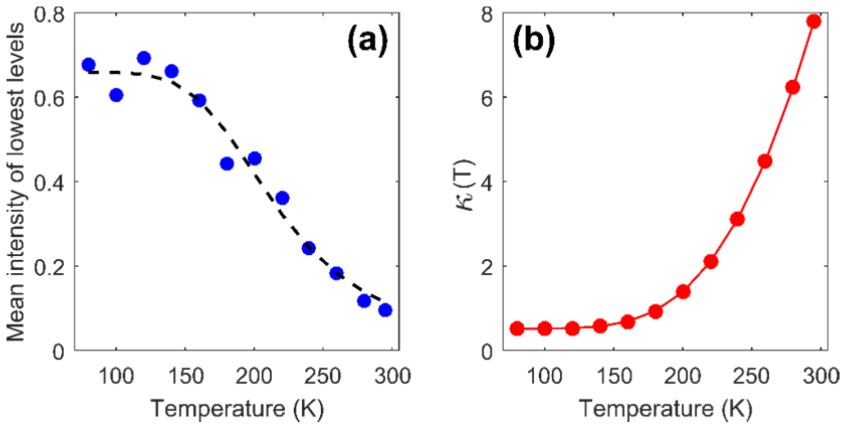

Supplementary Figure 27: Estimation of $\kappa(T)$. Panel (a) shows the mean of the lowest intensity levels extracted from the normalized blinking transients at different temperatures. An activation law fit (dashed line) was applied to capture the temperature dependent slope. Panel (b) shows the resulting function $\kappa(T)$ used in our model.

Consideration of time-averaging effects in the simulation

In our simulations, random switching times are generated based on exponential probability distributions defined by the characteristic switching times $\tau_{\mathrm{active}} = k_{\mathrm{a\to p}}^{-1}$ and $\tau_{\mathrm{passive}} = k_{\mathrm{p\to a}}^{-1}$. According to Equation 3 in the main text, the characteristic switching times depend on temperature and on the height of the energetic barrier E_{ap} and E_{pa}, respectively:

$$\tau_{\mathrm{active}} = \left[k_0 \exp\left(-\frac{E_{\mathrm{ap}}}{k_{\mathrm{B}}T}\right)\right]^{-1} \text{ and } \tau_{\mathrm{passive}} = \left[k_0 \exp\left(-\frac{E_{\mathrm{pa}}}{k_{\mathrm{B}}T}\right)\right]^{-1} \quad (12)$$

The general slope of the switching rates as function of temperature is presented in Figure 5(b) in the main text. From the plot it becomes obvious that there is a strong temperature dependence of the switching rates and with increasing temperature, they can become very large, which means that the switching times become very short. At some point, such events become too fast to be observed as blinking due to the limited integration time of the CCD camera. In such cases, the intensity fluctuations do not have to be considered explicitly in the simulations, which is essential to save computing time. The goal of this section is to find a reasonable value for a minimum switching time, below which the influence of a quencher can be considered as static due to the fast switching.

To study the time-averaging effect, we simulated blinking transients with time steps of 10 µs and averaged these transients in a next step according to the 50 ms integration time used in experiment. Similar to the experiment, the overall length of the simulated intensity transients was 100 s. As shown in Supplementary Figure 28(a), we varied the characteristic switching times $\tau = \tau_{\mathrm{active}} = \tau_{\mathrm{passive}}$ from 100 ms to 100 µs. With decreasing switching time, the blinking appears more and more as flickering and clearly defined 'on' and 'off' events are already largely absent for characteristic switching times below 30 ms. Accordingly, the blinking amplitudes reduce with decreasing switching time, as presented in Supplementary Figure 28(b-c). For a switching time of 0.3 ms, we find for example that the fraction of intensity jumps with amplitudes larger than 0.2 is smaller than 10^{-3}. Based on this analysis, we determine that if either τ_{active} or τ_{passive} is below a value of 0.3 ms, the quenchers are treated as static, because the residence times in either the active or the passive state become virtually too fast to be detected experimentally.

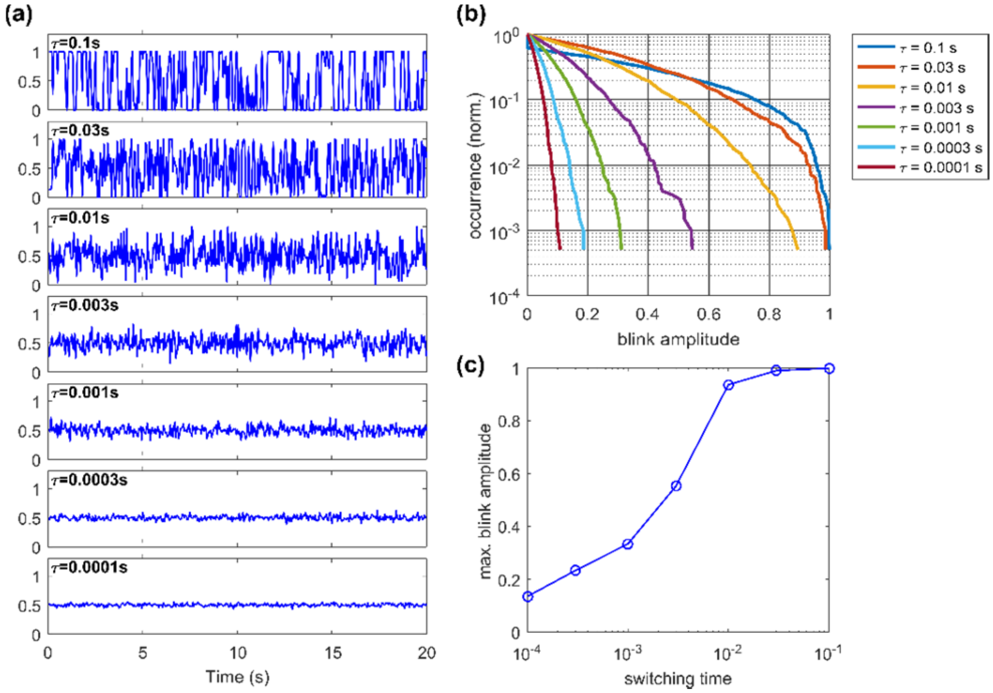

Supplementary Figure 28: Time-averaging effect for different switching times. Panel (a) shows how simulated blinking transients with different characteristic switching times $\tau = \tau_{\mathrm{active}} = \tau_{\mathrm{passive}}$ appear when the experimental integration time of 50 ms is considered. Panel (b) shows plots of the complementary cumulative distribution function of the intensity jumps extracted from the blinking transients presented in (a). Panel (c) shows a decrease of the maximum blinking amplitudes due to time-averaging.

Although these fast or 'quasi-static' quenchers do not cause remarkable intensity fluctuations, their influence on the overall PL intensity needs to be considered. In order to account for such influences in the simulations, we split the calculation of the PL intensity transients $\Phi(T,t)$ into two steps. First, we consider only the influence of those quenchers, which are slowly fluctuating and thus lead to time-dependent modulations of the PL intensity. According to Equation 2 in the main text, the PL intensity is given by:

$$\Phi(T,t) = \frac{\Phi_0(T)}{1 + \kappa \sum_{i=1}^{N} Q_i(T,t)} \tag{13}$$

Here, κ denotes the ratio between the fluctuating and the static decay rates, N is the number of quenchers and $Q_i(T,t)$ contains the information about the switching state of the fluctuating quencher i. The presence of an additional quasi-static quencher will lead to further reduction of $\Phi(T,t)$. The resulting PL intensity can be calculated by forming the weighted arithmetic mean between the PL intensity without the fast quencher $\Phi(T,t)$ and the PL intensity $\Phi'(T,t)$ that would result, if this quencher was permanently active:

$$\Phi'(T,t) = \frac{\Phi_0(T)}{1 + \kappa \sum_{i=1}^{N} Q_i(T,t) + \kappa} \tag{14}$$

The actual contribution of the quasi-static quencher is, however, smaller because it switches between active and passive state. Considering the characteristic switching times ($\tau_{\mathrm{a}} = \tau_{\mathrm{active}}$ and $\tau_{\mathrm{p}} = \tau_{\mathrm{passive}}$) of the quasi-static quencher, the resulting PL intensity is:

$$\Phi_1(t) = \frac{\tau_{\mathrm{p}}}{\tau_{\mathrm{a}} + \tau_p} \cdot \Phi(t) + \frac{\tau_{\mathrm{a}}}{\tau_{\mathrm{a}} + \tau_{\mathrm{p}}} \cdot \Phi'(t) \tag{15}$$

This equation can be rearranged to

$$\Phi_1(T,t) = \Phi(T,t) \cdot \left(1 - \frac{\tau_\mathrm{a}}{\tau_\mathrm{a} + \tau_\mathrm{p}} \cdot \frac{\kappa \cdot \Phi(T,t)}{1 + \kappa \cdot \Phi(T,t)}\right) \tag{16}$$

If more than one quasi-static quenchers are present, we use an iterative approach to calculate the PL intensity:

$$\Phi_\mathrm{n}(T,t) = \Phi_\mathrm{n-1}(T,t) \cdot \left(1 - \frac{\tau_\mathrm{a,n}}{\tau_\mathrm{a,n} + \tau_\mathrm{p,n}} \cdot \frac{\kappa \cdot \Phi_\mathrm{n-1}(T,t)}{1 + \kappa \cdot \Phi_\mathrm{n-1}(T,t)}\right) \tag{17}$$

Supplementary References

1. Tian, Y. & Scheblykin, I. G. Artifacts in absorption measurements of organometal halide perovskite materials: What are the real spectra? *J. Phys. Chem. Lett.* **6,** 3466–3470 (2015).
2. Milot, R. L., Eperon, G. E., Snaith, H. J., Johnston, M. B. & Herz, L. M. Temperature-Dependent Charge-Carrier Dynamics in $CH_3NH_3PbI_3$ Perovskite Thin Films. *Adv. Funct. Mater.* **25,** 6218–6227 (2015).
3. Tian, Y. *et al.* Giant photoluminescence blinking of perovskite nanocrystals reveals single-trap control of luminescence. *Nano Lett.* **15,** 1603–1608 (2015).
4. Xing, G. *et al.* Long-range balanced electron- and hole-transport lengths in organic-inorganic $CH_3NH_3PbI_3$. *Science* **342,** 344–7 (2013).
5. Leguy, A. M. A. *et al.* Experimental and theoretical optical properties of methylammonium lead halide perovskites. *Nanoscale* **8,** 6317–6327 (2016).
6. Shirayama, M. *et al.* Optical Transitions in Hybrid Perovskite Solar Cells: Ellipsometry, Density Functional Theory, and Quantum Efficiency Analyses for $CH_3NH_3PbI_3$. *Phys. Rev. Appl.* **5,** 014012 (2016).
7. Jiang, Y. *et al.* Temperature dependent optical properties of $CH_3NH_3PbI_3$ perovskite by spectroscopic ellipsometry. *Appl. Phys. Lett.* **108,** 061905 (2016).
8. Johnston, M. B. & Herz, L. M. Hybrid Perovskites for Photovoltaics: Charge-Carrier Recombination, Diffusion, and Radiative Efficiencies. *Acc. Chem. Res.* **49,** 146–154 (2016).
9. Tian, Y., Halle, J., Wojdyr, M., Sahoo, D. & Scheblykin, I. G. Quantitative measurement of fluorescence brightness of single molecules. *Methods Appl. Fluoresc.* **2,** 035003 (2014).
10. Fang, H. H. *et al.* Photophysics of organic-inorganic hybrid lead iodide perovskite single crystals. *Adv. Funct. Mater.* **25,** 2378–2385 (2015).
11. Yamada, Y. *et al.* Dynamic Optical Properties of $CH_3NH_3PbI_3$ Single Crystals As Revealed by One- and Two-Photon Excited Photoluminescence Measurements. *J. Am. Chem. Soc.* **137,** 10456–10459 (2015).
12. He, H. *et al.* Exciton localization in solution-processed organolead trihalide perovskites. *Nat. Commun.* **7,** 10896 (2016).
13. Zhao, Y.-C. *et al.* Quantification of light-enhanced ionic transport in lead iodide perovskite thin films and its solar cell applications. *Light Sci. Appl.* **6,** e16243 (2016).
14. Galkowski, K. *et al.* Determination of the exciton binding energy and effective masses for methylammonium and formamidinium lead tri-halide perovskite semiconductors. *Energy Environ. Sci.* **9,** 962–970 (2016).
15. Ziffer, M. E., Mohammed, J. C. & Ginger, D. S. Electroabsorption Spectroscopy Measurements of the Exciton Binding Energy, Electron-Hole Reduced Effective Mass, and Band Gap in the Perovskite CH3NH3PbI3. *ACS Photonics* **3,** 1060–1068 (2016).
16. Wen, X. *et al.* Mobile Charge-Induced Fluorescence Intermittency in Methylammonium Lead Bromide Perovskite. *Nano Lett.* **15,** 4644–4649 (2015).
17. Halder, A., Pathoor, N., Chowdhury, A. & Sarkar, S. K. Photoluminescence Flickering of Micron Sized Crystals of Methylammonium Lead Bromide: Effect of Ambience and Light Exposure. *J. Phys. Chem. C* **122,** 15133–15139 (2018).
18. Thomsson, D. *et al.* Cyclodextrin insulation prevents static quenching of conjugated polymer fluorescence at the single molecule level. *Small* **9,** 2619–2627 (2013).

19. Merdasa, A. *et al.* ‘Supertrap’ at Work: Extremely Efficient Nonradiative Recombination Channels in $MAPbI_3$ Perovskites Revealed by Luminescence Super-Resolution Imaging and Spectroscopy. *ACS Nano* **11,** 5391–5404 (2017).
